# Supplementary material for: First Experimental Evidence for Reversibility of Ammonia Loss from Asparagine
Source: Int J Mol Sci. 2022 Jul 28;23(15):8371. doi: 10.3390/ijms23158371 (PMC9368827; doi:10.3390/ijms23158371)
Supplement: Supplementary file 1 [file ijms-23-08371-s001.zip › ijms-1804868-supplementary.pdf]

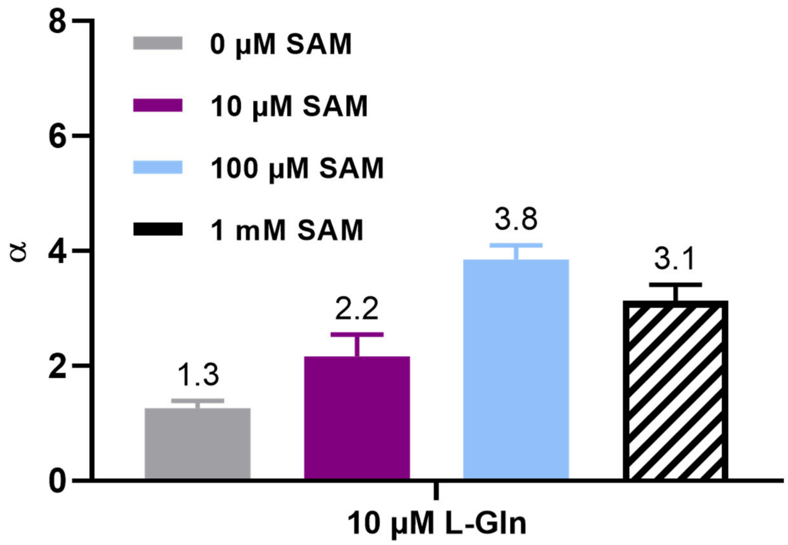

Figure S1. **The effect of *S*-adenosylmethionine (SAM) with different concentrations on protein succinimide/isoaspartate ammonia ligase (PSIAL) activity when adding 10 μM glutamine (Gln).**
